# 1-Allyl-3-benzyl-1*H*-benzimidazol-2(3*H*)-one

**DOI:** 10.1107/S1600536813023568

**Published:** 2013-08-31

**Authors:** Youssef Kandri Rodi, Amal Haoudi, Frédéric Capet, Ahmed Mazzah, El Mokhtar Essassi, Lahcen El Ammari

**Affiliations:** a Laboratoire de Chimie Organique Appliquée, Université Sidi Mohamed Ben Abdallah, Faculté des Sciences et Techniques, Route d’Immouzzer, BP 2202 Fès, Morocco; b Unité de Catalyse et de Chimie du Solide (UCCS), UMR 8181, Ecole Nationale Supérieure de Chimie de Lille, France; c USR 3290 Miniaturisation pour l’Analyse, la Synthèse et la Protéomique, 59655 Villeneuve d’Ascq Cedex, Université Lille 1, France; d Laboratoire de Chimie Organique Hétérocyclique, URAC 21, Pôle de Compétences Pharmacochimie, Université Mohammed V-Agdal, BP 1014 Avenue, Ibn Batouta, Rabat, Morocco; e Laboratoire de Chimie du Solide Appliquée, Faculté des Sciences, Université Mohammed V-Agdal, Avenue Ibn Battouta, BP 1014, Rabat, Morocco

## Abstract

In the title compound, C_17_H_16_N_2_O, the fused benzimidazol-2(3*H*)-one system is essentially planar, the largest deviation from the mean plane being 0.006 (2) Å for the carbonyl C atom. Its mean plane is almost perpendicular to the benzyl plane and to the allyl group, making dihedral angles of 80.6 (1) and 77.4 (3)°, respectively. The benzyl group and the allyl subsituent lie on opposite sides of the fused ring system. In the crystal, mol­ecules are linked by bifurcated C—H⋯O hydrogen bonds in which the carbonyl O atom acts as accepter to two aromatic C—H groups, forming a two-dimensional network parallel to (001).

## Related literature

For the biological activity of benzimidazole derivatives, see: Gravatt *et al.* (1994 ▶); Soderlind *et al.* (1999 ▶); Bouwman *et al.* (1990 ▶) and for potential applications in the treatment of some diseases, see: Zhu *et al.* (2008 ▶); Ogino *et al.* (2008 ▶); Shah *et al.* (2008 ▶). For their use as inter­mediates in chemical synthesis, see: Bai *et al.* (2001 ▶). For similar compounds, see: Belaziz *et al.* (2012 ▶, 2013 ▶).
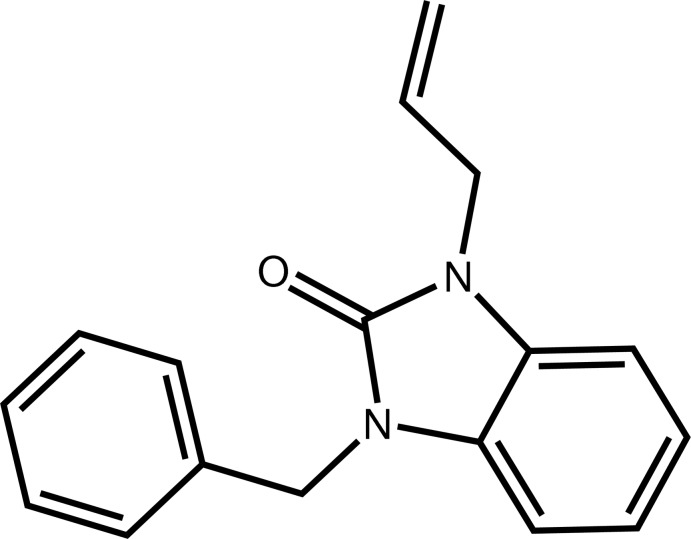


## Experimental

### 

#### Crystal data

C_17_H_16_N_2_O
*M* *_r_* = 264.32
Triclinic, 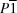

*a* = 9.0667 (2) Å
*b* = 9.3922 (2) Å
*c* = 9.6486 (2) Å
α = 94.218 (1)°
β = 113.543 (1)°
γ = 106.265 (1)°
*V* = 706.87 (3) Å^3^
*Z* = 2
Mo *K*α radiation
μ = 0.08 mm^−1^
*T* = 296 K
0.43 × 0.20 × 0.16 mm

#### Data collection

Bruker APEXII CCD diffractometer
Absorption correction: multi-scan (*SADABS*; Bruker, 2009 ▶) *T*_min_ = 0.967, *T*_max_ = 0.988
12670 measured reflections
3494 independent reflections
2573 reflections with *I* > 2σ(*I*)
*R*_int_ = 0.021

#### Refinement

*R*[*F* ^2^ > 2σ(*F* ^2^)] = 0.047
*wR*(*F* ^2^) = 0.141
*S* = 1.06
3494 reflections
181 parameters
H-atom parameters constrained
Δρ_max_ = 0.18 e Å^−3^
Δρ_min_ = −0.15 e Å^−3^

### 

Data collection: *APEX2* (Bruker, 2009 ▶); cell refinement: *SAINT-Plus* (Bruker, 2009 ▶); data reduction: *SAINT-Plus*; program(s) used to solve structure: *SHELXS97* (Sheldrick, 2008 ▶); program(s) used to refine structure: *SHELXL97* (Sheldrick, 2008 ▶); molecular graphics: *ORTEP-3 for Windows* (Farrugia, 2012 ▶); software used to prepare material for publication: *PLATON* (Spek, 2009 ▶) and *publCIF* (Westrip, 2010 ▶).

## Supplementary Material

Crystal structure: contains datablock(s) I. DOI: 10.1107/S1600536813023568/im2438sup1.cif

Structure factors: contains datablock(s) I. DOI: 10.1107/S1600536813023568/im2438Isup2.hkl

Supplementary material file. DOI: 10.1107/S1600536813023568/im2438Isup3.cml

Additional supplementary materials:  crystallographic information; 3D view; checkCIF report

## Figures and Tables

**Table 1:** Hydrogen-bond geometry (Å, °)

*D*—H⋯*A*	*D*—H	H⋯*A*	*D*⋯*A*	*D*—H⋯*A*
C3—H3⋯O1^i^	0.93	2.48	3.405 (2)	173
C14—H14⋯O1^ii^	0.93	2.56	3.330 (2)	141

Symmetry codes: (i) 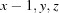; (ii) 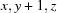.

**Fig. 1.**
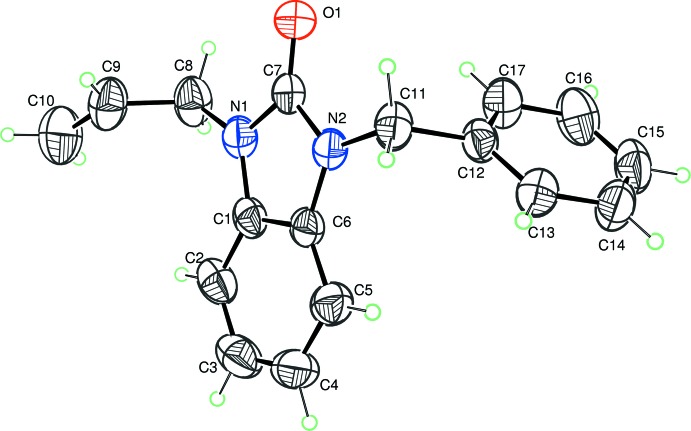
: Molecular structure of the title compound with the atom-labelling scheme. Displacement ellipsoids are drawn at the 50% probability level. H atoms are represented as small circles.

**Fig. 2.**
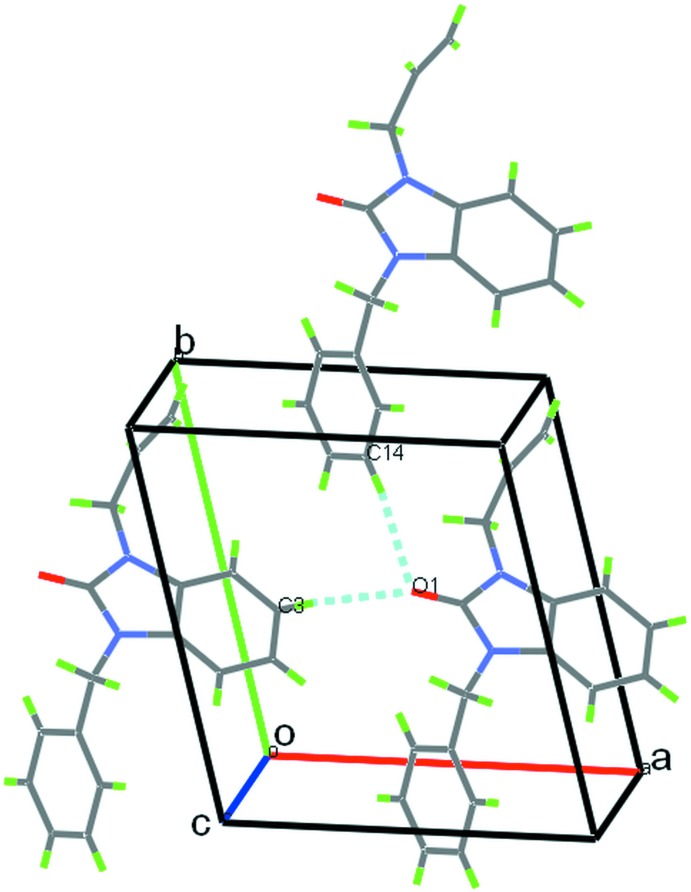
: Intermolecular interactions in the title compound building a two-dimensional network. Hydrogen bonds are shown as dashed blue lines.

## supplementary crystallographic information

### 1. Comment

Functionalized benzimidazoles represent an important class of N-containing heterocyclic compounds and have received considerable attention in recent times because of their applications as antiulcers, antihypertensives, antivirals, antifungals, anticancers and antihistamines among others (Gravatt *et al.*, 1994; Soderlind *et al.*, 1999). They are also important intermediates in many organic reactions (Bai *et al.*, 2001) and act as ligands to transition metals for modelling biological systems (Bouwman *et al.*, 1990). In addition, the treatment potency of benzimidazoles in diseases such as ischemia–reperfusion injury (Zhu *et al.*, 2008), hypertension (Ogino *et al.*, 2008), obesity (Shah *et al.*, 2008) *etc*. have been recently reported.

In continuation of our research work devoted to the development of substituted benzimidazol-2-one derivatives (Belaziz *et al.*, 2012, 2013), we reported a synthesis of new benzimidazol-2-one derivative differently substituted by action of allylbromide with 1-benzyl -1*H*-benzo[*d*]imidazol-2(3*H*)-one in the presence of a catalytic quantity of tetra-n-butylammonium bromide under mild conditions to obtain disubstituted compound (Scheme 1).

The molecule of title compound, 1-allyl-3-(benzyl)-1*H*-benzo[*d*]imidazol-2(3*H*)- one, is built up from two fused five- and six-membered rings linked to a benzyl substituent and an allyl group as shown in Fig. 1. The fused rings system (N1, N2, C1-C7) is essentially planar with the largest deviation from the mean plane being -0.006 (2) A° at C5 atom. The benzyl group and the allyl group are almost perpendicular to the benzo[*d*]imidazol-2(3*H*)-one as indicated by the dihedral angles of 80.6 (1) and 77.4 (3) °, respectively.

In the crystal, the molecules are linked by C3–H3···O1 and C14–H14···O1 hydrogen bonds in the way to build two-dimensional network as shown in Fig. 2 and Table 2.

### 2. Experimental

To 1*H*-benzo[*d*]imidazol-2(3*H*)-one (0.2 g, 1.49 mmol), potassium carbonate (0.41 g, 2.98 mmol) and tetra-n-butylammonium bromide (0.05 g, 0.15 mmol) in DMF (20 ml) was added benzylchloride (0.22 g, 1.79 mmol). Stirring was continued at room temperature for 6 h. The resulting salt was removed by filtration and the filtrate concentrated under reduced pressure. The residue was separated by chromatography on a column of silica gel with ethyl acetate/hexane (1/2) as eluent. The compound was recrystallized from ethanol to give colorless crystals. To the compound obtained (1-benzyl-benzimidazol-2-one (0.15 g, 0.67 mmol) was added allylbromide (0.10 g, 0.87 mmol) in DMF (10 ml), and potassium carbonate (0.18 g, 1.33 mmol) and tetra-n-butylammonium bromide (0.02 g, 0.07 mmol). Stirring was continued at room temperature for 12 h. The formed salt was removed by filtration and the filtrate concentrated under reduced pressure. The residue was separated by chromatography on a column of silica gel with ethyl acetate/hexane (1/1) as eluent (yield 82%, based on the intermediate). The title compound was recrystallized from dichloromethane/hexane to give colourless crystals.

### 3. Refinement

All H atoms could be located in a difference Fourier map. However, they were placed in calculated positions with C—H = 0.93 Å (aromatic, olefinic), and C—H = 0.97 Å (methylene) and refined as riding on their parent atoms with *U*_iso_(H) = 1.2 *U*_eq_ (C).

### Crystal data

**Table:**

C_17_H_16_N_2_O	*Z* = 2
*M**_r_* = 264.32	*F*(000) = 280
Triclinic, *P*1	*D*_x_ = 1.242 Mg m^−^^3^
Hall symbol: -P 1	Mo *K*α radiation, λ = 0.71073 Å
*a* = 9.0667 (2) Å	Cell parameters from 3494 reflections
*b* = 9.3922 (2) Å	θ = 2.3–28.3°
*c* = 9.6486 (2) Å	µ = 0.08 mm^−^^1^
α = 94.218 (1)°	*T* = 296 K
β = 113.543 (1)°	Irregular shape, colourless
γ = 106.265 (1)°	0.43 × 0.20 × 0.16 mm
*V* = 706.87 (3) Å^3^	

### Data collection

**Table:**

Bruker APEXII CCD diffractometer	3494 independent reflections
Radiation source: microfocus source	2573 reflections with *I* > 2σ(*I*)
Graphite monochromator	*R*_int_ = 0.021
φ and ω scans	θ_max_ = 28.3°, θ_min_ = 2.3°
Absorption correction: multi-scan (*SADABS*; Bruker, 2009)	*h* = −12→12
*T*_min_ = 0.967, *T*_max_ = 0.988	*k* = −12→12
12670 measured reflections	*l* = −12→12

### Refinement

**Table:**

Refinement on *F*^2^	Primary atom site location: structure-invariant direct methods
Least-squares matrix: full	Secondary atom site location: difference Fourier map
*R*[*F*^2^ > 2σ(*F*^2^)] = 0.047	Hydrogen site location: inferred from neighbouring sites
*wR*(*F*^2^) = 0.141	H-atom parameters constrained
*S* = 1.06	*w* = 1/[σ^2^(*F*_o_^2^) + (0.0617*P*)^2^ + 0.1232*P*] where *P* = (*F*_o_^2^ + 2*F*_c_^2^)/3
3494 reflections	(Δ/σ)_max_ < 0.001
181 parameters	Δρ_max_ = 0.18 e Å^−^^3^
0 restraints	Δρ_min_ = −0.15 e Å^−^^3^

### Special details

**Table:**

Geometry. All s.u.'s (except the s.u. in the dihedral angle between two l.s. planes) are estimated using the full covariance matrix. The cell s.u.'s are taken into account individually in the estimation of s.u.'s in distances, angles and torsion angles; correlations between s.u.'s in cell parameters are only used when they are defined by crystal symmetry. An approximate (isotropic) treatment of cell s.u.'s is used for estimating s.u.'s involving l.s. planes.
Refinement. Refinement of *F*^2^ against all reflections. The weighted *R*-factor *wR* and goodness of fit *S* are based on *F*^2^, conventional *R*-factors *R* are based on *F*, with *F* set to zero for negative *F*^2^. The threshold expression of *F*^2^ > σ(*F*^2^) is used only for calculating *R*-factors(gt) *etc*. and is not relevant to the choice of reflections for refinement. *R*-factors based on *F*^2^ are statistically about twice as large as those based on *F*, and *R*- factors based on all data will be even larger.

### Fractional atomic coordinates and isotropic or equivalent isotropic displacement parameters (Å^2^)

**Table:**

	*x*	*y*	*z*	*U*_iso_*/*U*_eq_	
C1	0.08458 (17)	0.53012 (15)	0.75007 (15)	0.0503 (3)	
C2	−0.0580 (2)	0.4975 (2)	0.77755 (18)	0.0652 (4)	
H2	−0.0800	0.4235	0.8325	0.078*	
C3	−0.1663 (2)	0.5780 (2)	0.7211 (2)	0.0785 (5)	
H3	−0.2638	0.5575	0.7376	0.094*	
C4	−0.1336 (2)	0.6887 (2)	0.6404 (2)	0.0778 (5)	
H4	−0.2098	0.7410	0.6034	0.093*	
C5	0.0111 (2)	0.72378 (18)	0.61297 (19)	0.0636 (4)	
H5	0.0338	0.7989	0.5594	0.076*	
C6	0.11833 (17)	0.64210 (14)	0.66878 (15)	0.0484 (3)	
C7	0.33127 (18)	0.54248 (15)	0.73733 (17)	0.0524 (3)	
C8	0.2415 (2)	0.35712 (18)	0.88407 (19)	0.0710 (5)	
H8A	0.2268	0.3845	0.9755	0.085*	
H8B	0.3582	0.3588	0.9181	0.085*	
C9	0.1241 (3)	0.20112 (19)	0.8030 (2)	0.0777 (5)	
H9	0.1257	0.1609	0.7128	0.093*	
C10	0.0209 (3)	0.1174 (3)	0.8466 (4)	0.1171 (9)	
H10A	0.0154	0.1535	0.9362	0.141*	
H10B	−0.0490	0.0201	0.7889	0.141*	
C11	0.3572 (2)	0.74859 (15)	0.59174 (18)	0.0576 (4)	
H11A	0.2751	0.7508	0.4904	0.069*	
H11B	0.4401	0.7099	0.5778	0.069*	
C12	0.44798 (18)	0.90823 (15)	0.68814 (16)	0.0507 (3)	
C13	0.4105 (2)	1.02876 (17)	0.6275 (2)	0.0635 (4)	
H13	0.3285	1.0118	0.5261	0.076*	
C14	0.4935 (3)	1.17505 (18)	0.7159 (3)	0.0762 (5)	
H14	0.4671	1.2557	0.6738	0.091*	
C15	0.6135 (3)	1.2009 (2)	0.8642 (3)	0.0798 (6)	
H15	0.6686	1.2991	0.9236	0.096*	
C16	0.6534 (3)	1.0830 (2)	0.9259 (2)	0.0854 (6)	
H16	0.7360	1.1011	1.0273	0.102*	
C17	0.5712 (2)	0.93636 (19)	0.8379 (2)	0.0709 (5)	
H17	0.5994	0.8565	0.8803	0.085*	
N1	0.21567 (15)	0.47062 (13)	0.79022 (14)	0.0546 (3)	
N2	0.26911 (15)	0.64676 (12)	0.66155 (14)	0.0504 (3)	
O1	0.46264 (14)	0.51931 (12)	0.75495 (15)	0.0703 (3)	

### Atomic displacement parameters (Å^2^)

**Table:**

	*U*^11^	*U*^22^	*U*^33^	*U*^12^	*U*^13^	*U*^23^
C1	0.0468 (7)	0.0456 (7)	0.0449 (7)	0.0041 (6)	0.0168 (6)	−0.0037 (5)
C2	0.0565 (9)	0.0695 (10)	0.0581 (9)	0.0033 (7)	0.0286 (7)	−0.0032 (7)
C3	0.0530 (9)	0.0892 (13)	0.0808 (12)	0.0110 (9)	0.0317 (9)	−0.0143 (10)
C4	0.0553 (10)	0.0799 (12)	0.0836 (12)	0.0281 (9)	0.0168 (9)	−0.0067 (10)
C5	0.0600 (9)	0.0563 (9)	0.0633 (9)	0.0209 (7)	0.0168 (7)	0.0043 (7)
C6	0.0462 (7)	0.0418 (6)	0.0467 (7)	0.0087 (5)	0.0164 (6)	−0.0024 (5)
C7	0.0488 (8)	0.0387 (6)	0.0586 (8)	0.0074 (6)	0.0193 (6)	0.0016 (6)
C8	0.0755 (11)	0.0578 (9)	0.0593 (9)	0.0110 (8)	0.0165 (8)	0.0192 (7)
C9	0.0935 (13)	0.0534 (9)	0.0736 (11)	0.0171 (9)	0.0287 (10)	0.0196 (8)
C10	0.120 (2)	0.0697 (13)	0.162 (2)	0.0150 (13)	0.0698 (18)	0.0428 (15)
C11	0.0682 (9)	0.0451 (7)	0.0594 (8)	0.0098 (7)	0.0352 (7)	0.0057 (6)
C12	0.0544 (8)	0.0424 (7)	0.0567 (8)	0.0078 (6)	0.0322 (7)	0.0066 (6)
C13	0.0704 (10)	0.0526 (8)	0.0670 (9)	0.0180 (7)	0.0316 (8)	0.0126 (7)
C14	0.0904 (13)	0.0455 (8)	0.1024 (15)	0.0198 (8)	0.0541 (12)	0.0139 (9)
C15	0.0848 (13)	0.0490 (9)	0.0948 (14)	−0.0039 (8)	0.0512 (11)	−0.0105 (9)
C16	0.0789 (12)	0.0732 (12)	0.0676 (11)	−0.0026 (9)	0.0191 (9)	−0.0043 (9)
C17	0.0726 (11)	0.0556 (9)	0.0693 (10)	0.0109 (8)	0.0237 (9)	0.0128 (8)
N1	0.0539 (7)	0.0447 (6)	0.0559 (7)	0.0095 (5)	0.0199 (5)	0.0109 (5)
N2	0.0508 (6)	0.0381 (5)	0.0604 (7)	0.0103 (5)	0.0262 (5)	0.0074 (5)
O1	0.0533 (6)	0.0580 (6)	0.0960 (9)	0.0210 (5)	0.0287 (6)	0.0116 (6)

### Geometric parameters (Å, º)

**Table:**

C1—C2	1.378 (2)	C9—C10	1.274 (3)
C1—N1	1.3824 (19)	C9—H9	0.9300
C1—C6	1.396 (2)	C10—H10A	0.9300
C2—C3	1.371 (3)	C10—H10B	0.9300
C2—H2	0.9300	C11—N2	1.4503 (18)
C3—C4	1.381 (3)	C11—C12	1.5109 (19)
C3—H3	0.9300	C11—H11A	0.9700
C4—C5	1.396 (3)	C11—H11B	0.9700
C4—H4	0.9300	C12—C13	1.376 (2)
C5—C6	1.373 (2)	C12—C17	1.377 (2)
C5—H5	0.9300	C13—C14	1.385 (2)
C6—N2	1.3853 (18)	C13—H13	0.9300
C7—O1	1.2181 (17)	C14—C15	1.359 (3)
C7—N1	1.3771 (19)	C14—H14	0.9300
C7—N2	1.3773 (18)	C15—C16	1.364 (3)
C8—N1	1.4572 (19)	C15—H15	0.9300
C8—C9	1.478 (2)	C16—C17	1.386 (2)
C8—H8A	0.9700	C16—H16	0.9300
C8—H8B	0.9700	C17—H17	0.9300
			
C2—C1—N1	131.78 (15)	H10A—C10—H10B	120.0
C2—C1—C6	121.12 (15)	N2—C11—C12	112.61 (11)
N1—C1—C6	107.10 (12)	N2—C11—H11A	109.1
C3—C2—C1	117.73 (17)	C12—C11—H11A	109.1
C3—C2—H2	121.1	N2—C11—H11B	109.1
C1—C2—H2	121.1	C12—C11—H11B	109.1
C2—C3—C4	121.36 (17)	H11A—C11—H11B	107.8
C2—C3—H3	119.3	C13—C12—C17	118.60 (14)
C4—C3—H3	119.3	C13—C12—C11	120.70 (14)
C3—C4—C5	121.50 (17)	C17—C12—C11	120.70 (14)
C3—C4—H4	119.2	C12—C13—C14	120.70 (17)
C5—C4—H4	119.2	C12—C13—H13	119.6
C6—C5—C4	116.84 (17)	C14—C13—H13	119.6
C6—C5—H5	121.6	C15—C14—C13	120.04 (17)
C4—C5—H5	121.6	C15—C14—H14	120.0
C5—C6—N2	131.80 (14)	C13—C14—H14	120.0
C5—C6—C1	121.45 (14)	C14—C15—C16	120.13 (16)
N2—C6—C1	106.75 (12)	C14—C15—H15	119.9
O1—C7—N1	126.96 (14)	C16—C15—H15	119.9
O1—C7—N2	126.92 (14)	C15—C16—C17	120.12 (18)
N1—C7—N2	106.13 (12)	C15—C16—H16	119.9
N1—C8—C9	114.03 (14)	C17—C16—H16	119.9
N1—C8—H8A	108.7	C12—C17—C16	120.40 (17)
C9—C8—H8A	108.7	C12—C17—H17	119.8
N1—C8—H8B	108.7	C16—C17—H17	119.8
C9—C8—H8B	108.7	C7—N1—C1	109.97 (12)
H8A—C8—H8B	107.6	C7—N1—C8	122.70 (14)
C10—C9—C8	125.3 (2)	C1—N1—C8	127.18 (14)
C10—C9—H9	117.4	C7—N2—C6	110.04 (12)
C8—C9—H9	117.4	C7—N2—C11	123.15 (12)
C9—C10—H10A	120.0	C6—N2—C11	126.76 (12)
C9—C10—H10B	120.0		
			
N1—C1—C2—C3	−179.88 (14)	C15—C16—C17—C12	−0.5 (3)
C6—C1—C2—C3	0.6 (2)	O1—C7—N1—C1	178.76 (14)
C1—C2—C3—C4	−0.4 (2)	N2—C7—N1—C1	−0.76 (15)
C2—C3—C4—C5	−0.1 (3)	O1—C7—N1—C8	2.9 (2)
C3—C4—C5—C6	0.6 (2)	N2—C7—N1—C8	−176.64 (12)
C4—C5—C6—N2	179.56 (14)	C2—C1—N1—C7	−179.26 (14)
C4—C5—C6—C1	−0.5 (2)	C6—C1—N1—C7	0.34 (15)
C2—C1—C6—C5	−0.1 (2)	C2—C1—N1—C8	−3.6 (2)
N1—C1—C6—C5	−179.75 (12)	C6—C1—N1—C8	175.99 (13)
C2—C1—C6—N2	179.87 (12)	C9—C8—N1—C7	−109.85 (18)
N1—C1—C6—N2	0.22 (14)	C9—C8—N1—C1	75.0 (2)
N1—C8—C9—C10	−121.4 (2)	O1—C7—N2—C6	−178.62 (14)
N2—C11—C12—C13	−122.16 (15)	N1—C7—N2—C6	0.90 (15)
N2—C11—C12—C17	58.5 (2)	O1—C7—N2—C11	−1.0 (2)
C17—C12—C13—C14	−0.7 (2)	N1—C7—N2—C11	178.55 (11)
C11—C12—C13—C14	179.94 (15)	C5—C6—N2—C7	179.26 (14)
C12—C13—C14—C15	0.0 (3)	C1—C6—N2—C7	−0.70 (14)
C13—C14—C15—C16	0.5 (3)	C5—C6—N2—C11	1.7 (2)
C14—C15—C16—C17	−0.3 (3)	C1—C6—N2—C11	−178.24 (12)
C13—C12—C17—C16	0.9 (3)	C12—C11—N2—C7	−102.99 (15)
C11—C12—C17—C16	−179.72 (16)	C12—C11—N2—C6	74.25 (18)

### Hydrogen-bond geometry (Å, º)

**Table:**

*D*—H···*A*	*D*—H	H···*A*	*D*···*A*	*D*—H···*A*
C3—H3···O1^i^	0.93	2.48	3.405 (2)	173
C14—H14···O1^ii^	0.93	2.56	3.330 (2)	141

Symmetry codes: (i) *x*−1, *y*, *z*; (ii) *x*, *y*+1, *z*.
